# Induction of resistance to potato virus Y strain NTN in potato plants through RNAi

**DOI:** 10.1080/13102818.2014.984968

**Published:** 2014-11-28

**Authors:** Nikolay Petrov, Mariya Stoyanova, Rayna Andonova, Atanaska Teneva

**Affiliations:** ^a^ Department of Plant Protection, Institute of Soil Science, Agro Technologies and Plant Protection “N. Pushkarov”, Sofia, Bulgaria; ^b^ Department of Plant Protection, Faculty of Agronomy, University of Forestry, Sofia, Bulgaria; ^c^ Department of Genetics and Breeding of Agricultural Crops, Faculty of Agronomy, University of Forestry, Sofia, Bulgaria

**Keywords:** RNAi, PVY^NTN^, potatoes

## Abstract

Potato viruses cause enormous economic loss in agriculture production. Potatoes can be infected by a number of different viruses that affect negatively the harvest and the tuber quality. Direct and effective drugs against plant virus diseases are still not available and control is only applied as agricultural measures and pesticides against virus vectors. Potato virus Y (PVY) is transmitted by aphids in non-persistent manner and on that account using insecticides to prevent spread of the infection is useless. Breeding of resistant plant cultivars proved to be not always a solution of the problem because of the fast evolution of the virus strains and the constantly growing group of recombinants. In this study, we have proposed a new way of controlling the virus by blocking replication and transmission through the plant by RNAi-based vaccination of potato seedlings with specific to viral HC-Pro gene siRNAs. Thus, PVY replication is decreased without altering the valuable qualities of the sensitive to the virus potato cultivars like Agria.

## Introduction

Potato virus Y (PVY) was first reported from Smith and was considered as a complex of virus isolates.[1] Potato virus C was first reported in 1930 [2,3] and it was the first one from a strain group, named later PVY^C^. The first report of PVY in Bulgaria was made by Kovachevski,[4,5] who established necrotic symptoms on pepper caused by this virus. Other strain group PVY^N^[6] was reported for the first time in 1935 in a tobacco field near experimental potato plants.[7] This strain group caused severe epidemics on the potato and tobacco plants in Europe in 1950.[8] РVY^O^ group, also called ordinary strains, was widely distributed and caused severe symptoms as mottling and leaf curling, necrosis on *Physalis floridana* and leaf spots on tobacco.[9] PVY^N^ strains inducted vein necrosis on tobacco, leaf spots on potatoes and necrosis on *P. floridana*.[9] PVY^NTN^ was first reported in Hungary in 1978.[10] PVY^NTN^ strain resembled РVY^N^, but induced necrotic ring spots on potato tubers. Nowadays, this strain was considered as a subgroup of PVY^−N^ group. PVY was identified as polyphagous in many plant species. Recently, the virus has been identified as a pathogen of the medicinal plant echinacea which is known as an extremely potent immunostimulant[11–13] and also of the essential oil-bearing plant coriander.[12]

**Figure 1.**
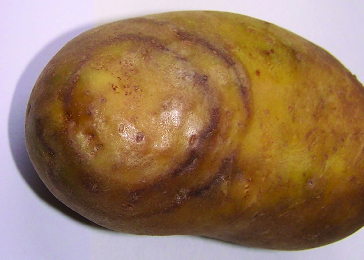
Concentric necrotic rings on potato tubers.

In Bulgaria, eight strains of PVY were distinguished in potatoes, tomatoes, pepper and tobacco – PVY^N/NTN^, PVY^N:O^, PVY^N^, PVY^NTN^, PVY^O^, PVY^Eu-NTN^, PVY^Na-NTN^ and PVY^C^, respectively.[14,15] Characteristic concentric necrotic rings in potato tubers were noticed from the Smolyan region (Figure 1) which later were identified as a PVY^NTN^ strain.[15]

Control of the virus has been very difficult. In most cases, the agricultural activities and vector control has been made to limit virus spread. A relatively useful effect of blocking viral replication by thermotherapy and electrotherapy was recently established,[16] but at the expense of reduced germination of tubers. Bion (benzo [1,2,3] thiadiazole-7-carbothioic acid-S-methyl ester or benzothiadiazole, BTH) and EXIN showed good results against PVY in tomatoes.[17] Better effect was achieved by gene silencing of the expression of the suppressor protein of PVY.[18,19]

Systemic spread of potyviruses included replication in initially infected epidermal or mesophylic cells, moving from cell to cell by the plasmodesmas then in the vascular tissues of the host plant and over long distances through the phloem following the distribution of photoassimilates.[20,21] The viral proteins responsible for the movement of the virus were necessary for the intracellular transfer of the virus by modifying plasmodesmatic channels so as to let the virus to move from cell to cell.[22] Potyviruses possess several multifunctional proteins involved in the movement of the virus – HC-Pro, Cl, 6k2, VPg and CP.[23] CP, HC-Pro and VPg proteins are involved in viral movement over large distances in phloem.[24] HC-Pro protein provides entry and exit of the virus from the vascular tissues of the host plant.[25] HC-Pro increases viral pathogenicity through suppression of post-transcriptional gene silencing (PTGS) in the host plant.[26] In the absence of functional HC-Pro, the viral RNA is targeted to the natural response of gene silencing of the host plant,[26] while its expression in transgenic plants suppresses PTGS prior to the occurrence of small interfering RNAs (siRNA).[27]

HC-Pro was a suppressor of intracellular gene silencing, but the signal was continuously expressed and exported outside the HC-Pro expressing cells, with the result that can reduce the level of viral spread in the healthy tissues of infected plants.[27]

The process of PTGS is initiated by double stranded RNAs (dsRNAs) that were produced during viral replication. The dsRNAs are recognized by the plant as a ‘non-own’ and subsequently cut by Dicer-like cellular enzymes to form siRNAs.[28] These molecules are the main component of the RNA gene silencing.[29] They initiated complementary-specific RNA degradation by forming a multicomponent enzyme RNA interference silencing complex (RISC), inducing RNA gene silencing that destroy cognate mRNAs.[30] Remarkable feature of the RNA gene silencing is its ability to spread both from cell to cell and over long distances causing systemic RNA silencing throughout the whole organism by complementary-specific signal silencing obtained after induction of RNA gene silencing in single cells.[31] In response, plant viruses encode proteins capable of suppressing RNA gene silencing.[31,32] The first reported viral suppressors of gene silencing were HC-Pro and 2b proteins encoded from potyviruses and cucumoviruses, respectively.[26,33] Potyviral HC-Pro was a multifunctional protein that participated in the transport of virions with aphids as well as in the movement of the virus in the plant and suppression of RNA-dependent gene silencing, established as a defence mechanism against viruses.[24,26]

There are still no effective substances for reduction of the viral infection, which is imperative for developing new approaches to block the replication of PVY.

## Materials and methods

The material consisted of:

- plants – 18 plant pots with potatoes cv. Agria;
- virus: PVY strain NTN obtained from potato cv. Desire from the virus collection of Institute of Soil Sciences, Agro Technologies and Plant Protection (ISSAPP);
- referent compounds: dsRNA for the S segment of Phi6 and siRNAs for the S segment of Phi6.

Mechanical inoculation of plants with PVY: the plants were inoculated as described by Noordam [34]. Prior to inoculation, the plants were placed in a room with low light (shading), sprinkled with water and the leaves were dusted with carborundum 400–600 meshes.

One gram of the symptomatic plant foliage was homogenized in 1 ml of cooled to 4 °C 0.1M potassium sodium phosphate buffer, pH 8.0 containing 0.2% Na_2_SO_3_ and 0.2% ascorbic acid. Inoculations were performed by gently rubbing the leaves with this homogenate. After 3–5 minutes, the plants were washed with water.

Serological diagnostic test: DAS-ELISA (Double Antibody Sandwich Еnzyme Linked Immunosorbent Assay): the analysis was conducted by the method of Clark and Adams [35]. We used a commercial kit of LOEWE (Biochemica GmbH, Germany). ELISA plates were loaded with antiserum (IgG) for PVY with dilutions in 0.05M carbonate buffer according to the instructions of the manufacturer. The samples were incubated for 4 hours at 37 °C, and the unbound components were washed out with phosphate buffered saline-tween (PBS-T) buffer for 5 minutes. All samples were grounded in extraction buffer containing 1% PVP (polyvinyl pyrrolidone) in a ratio of 1:10. The plates were incubated at 4 °C for 16 hours. Following the third wash step, alkaline–phosphatase conjugate for PVY was added and the plates were incubated for 4 hours at 37 °C. The substrate was *p*-nitrophenyl phosphate (*p*-nitrophenyl phosphate, Sigma) in diethanolamine buffer (pH 9.8) at a ratio of 1 mg/ml. The reaction proceeded in the light at room temperature and was stopped with 3N NaOH. The adsorption of the colour reaction was measured in a multifunctional detector (DTX 880) at a wavelength of 405 nm.

The positive samples had optical density (OD) over the threshold (cut-off) which was two times the value of the negative control.

RNA extraction from potatoes infected with PVY: extraction of total RNA was performed with RNEasy Plant Mini Kit (Qiagen, Germany). Extraction was carried out according to the instructions of the manufacturer.

*In vitro* system for the production of dsRNA: dsRNA was synthesized by a combination of *in vitro* transcription and replication of DNA template (according to Replicator RNAi Kit, Finnzymes, Finland). DNA template for synthesis of dsRNA was obtained by PCR using Phusion High-Fidelity DNA polymerase. Primers for the PCR were designed so that the resulting PCR fragment contained a target sequence (HC-Pro of PVY), flanked by T7 promoter sequences in the 5′ end and phi6 RdRP promoter sequences in the 3′ end. PCR DNA product was purified and transcribed by T7 viral RNA polymerase to ssRNA. The ssRNA was replicated to dsRNA by virus phi6 RdRP. The sequences of our designed primers were: HC-Pro dsRNA 1 (5′-TAA TAC GAC TCA CTA TAG GG TAG GAT TCT GTC GAA TGC CGA CAA TTT T -3′) and HC-Pro dsRNA 2 (5′-GGA AAA AAA TAC TGC AGA CCA ACT CTA TAA TGT TT -3′).

### Production of siRNAs

The PowerCut Dicer is a recombinant endoribonuclease from *Giardia intestinalis*. It cleaved dsRNA efficiently, producing fragments with a length of 25–27 nucleotides, yielding a pool of siRNAs.

## Results and discussion

**Figure 2.**
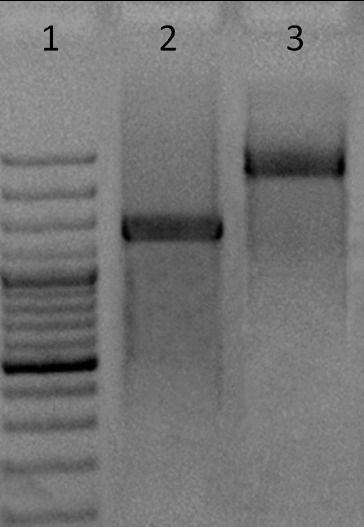
dsRNAs of the HC-Pro gene region of PVY and S segment of Phi6. (1) 100 bp DNA ladder (100/1000 bp); (2) dsRNAs of the HC-Pro gene region of PVY 1445 bp and (3) dsRNAs of S segment of Phi6 2948 bp.

*In vitro* system for generating dsRNA combined T7 RNA polymerase to synthesize ssRNA templates from PCR product template of the selected fragment of the PVY genome and viral Phi6 RdRP polymerase formed *de novo* initiation and synthesis of dsRNA from the used template ssRNA. Viral Phi6 RdRP-based system allowed the generation of dsRNAs to attack our target genetic sequence. This system was efficient, high quality and convenient method of obtaining high-quality dsRNAs from ssRNAs, such as PVY HC-Pro gene with length 1445 bp (Figure 2). From these specific to HC-Pro gene dsRNAs, we produced siRNAs with PowerCut Dicer.

**Figure 3.**
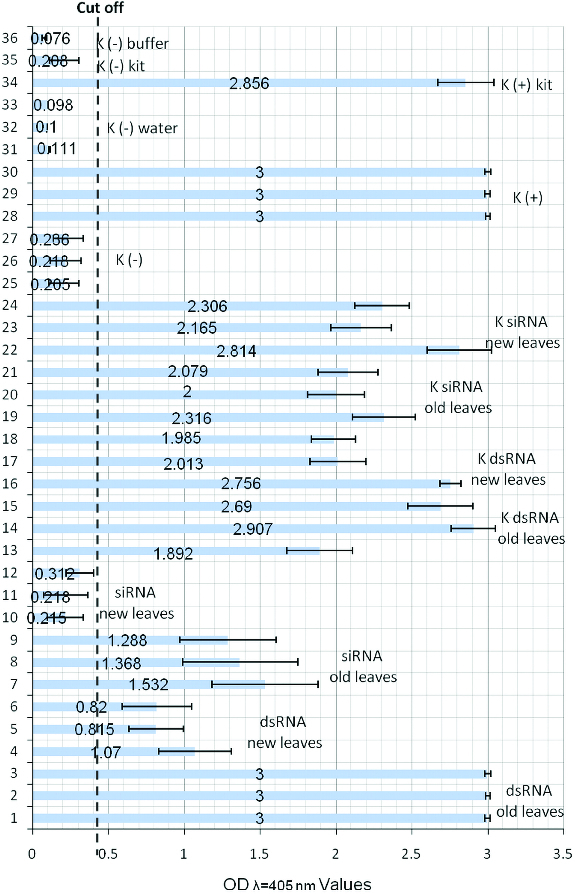
DAS-ELISA of potato plants after treatment with dsRNA and siRNAs. OD values of samples from: 1/3 – old leaves (at the moment of treatment and virus inoculation) of potato plants treated with HC-Pro dsRNAs and inoculated with PVY; 4/6 – new leaves (leaves grown after treatment) of potato plants treated with HC-Pro dsRNAs and inoculated with PVY; 7/9 – old leaves and 10/12 – new leaves of potato plants treated with HC-Pro siRNAs and inoculated with PVY; 13/15 – old leaves and 16/18 – new leaves of potato plants treated with control dsRNAs from the S segment of Phi 6 and inoculated with PVY; 19/21 – old leaves and 22/24 – new leaves of potato plants treated with control siRNAs from the S segment of Phi 6 and inoculated with PVY; 25/27 – control leaf samples of healthy potato plants not treated and not inoculated with virus; 28/30 – control leaf samples of potato plants inoculated with PVY and not treated; 31/33 – control leaf samples of healthy potato plants treated only with water; 34 – K+ control from the Kit; 35 – K− control from the Kit; 36 – buffer control.

Fourteen days after inoculation with dsRNAs and siRNAs and seven days after inoculation with PVY^NTN^ Agria plants were tested by DAS-ELISA with polyclonal serum IgG (LOEWE) for presence or absence of PVY viral infection in different plant parts (old leaves and newly grown leaves). We received high OD values of samples from old leaves of potato plants treated with HC-Pro dsRNAs and siRNAs and inoculated with PVY (Figure 3; for 1–3 and 7–9) which was a confirmation that PVY stayed in old parts of the plants despite treatment. OD values of the newly grown leaves of the same potato plants were over the cut-off for the potato plants treated with HC-Pro dsRNAs (Figure 3; 4–6) but under the cut-off for the potato plants treated with HC-Pro siRNAs (Figure 3; 10–12). These small OD values of the plants treated with HC-Pro siRNAs confirmed absence of PVY infection in the newly grown parts of potato plants due to the blocking of essential for virus replication HC-Pro gene region of PVY. The old infected leaves of these plants later defoliated and all new leaves grown after the inoculations (not treated) remained virus free. As controls in the experiment we used dsRNAs and siRNAs of S segment of bacteriophage Phi6. All treated plants with these unspecific for PVY dsRNAs and siRNAs remained infected with PVY (Figure 3; 13–24).

## Conclusions

Blocking the HC-Pro gene of PVY^NTN^ in newly grown leaves of potato plants cv. Agria was established. The old leaves remained infected but later defoliated leaving the plants virus free.

PTGS was induced in potato plants cv. Agria by specific siRNAs for HC-Pro region of PVY^NTN^ which effectively blocked the viral replication and the systemic spread of the virus.
